# Ketogenic diet as a systems-level immunometabolic sensitization strategy in cancer therapy: integrating metabolism, immune reprogramming, microbiome dynamics, and epigenetic regulation

**DOI:** 10.1007/s12032-026-03425-0

**Published:** 2026-09-25

**Authors:** Muhammad Shaheer Mannan, Muhammad Waqas Khan, Muhammad Abdul Haseeb Khan, Ahmed Javed, Syed Mujtaba Hussain, Hudda Adil

**Affiliations:** 1 Department of Internal Medicine, Marshfield Clinic, Marshfield, WI USA; 2 Department of Internal Medicine, Medical University of South Carolina, Charleston, SC USA; 3 Ayub Medical College, Abbottabad, Pakistan

**Keywords:** Ketogenic diet, Cancer immunometabolism, Immune reprogramming, Tumor microenvironment, Gut microbiome, Epigenetic regulation

## Abstract

The ketogenic diet (KD) is increasingly being recognized as more than a simple metabolic intervention in cancer therapy. Emerging evidence suggests that KD may function as a systems-level immunometabolic sensitization strategy capable of enhancing therapeutic responsiveness through interconnected biologic mechanisms. Modern cancer therapies, including chemotherapy, targeted therapy, radiotherapy, and immunotherapy, are often limited by tumor adaptability and immune suppression. Tumors shift metabolism via the Warburg effect and fuel switching, promoting resistance under hypoxic and nutrient stress. KD is a high-fat and low-carb eating plan that increases ketone bodies such as β-hydroxybutyrate while lowering glucose availability. Under ketogenic conditions, cancer cells reduce glycolytic flux, while immune cells can oxidise ketones to sustain mitochondrial function and effector activity. KD also remodels the gut microbiome and induces epigenetic regulation through histone deacetylase inhibition. These combined effects may enhance chemotherapy, radiotherapy, targeted therapy, and immunotherapy by inducing metabolic stress, improving immune cell fitness, and altering tumour microenvironment signaling. Importantly, KD may function not as a standalone treatment but as a therapy-sensitization platform. However, current evidence remains preliminary and heterogeneous, and further mechanistic investigations and well-designed prospective clinical trials are necessary to determine optimal patient selection and long-term safety.

## Introduction

### Limitations of current cancer therapies and metabolic–immune mismatch

Modern cancer therapies, including chemotherapy, targeted therapy, radiotherapy, and immunotherapy, are often limited by tumor adaptability and immune suppression. Tumors shift metabolism via the Warburg effect and fuel switching, promoting resistance under hypoxic and nutrient stress. This also contributes to lactic acidosis and redox imbalance, leading to T-cell exhaustion [1]. In our synthesis of this literature, this suggests a negative feedback loop that is created by tumour metabolic plasticity and immune exhaustion, which hampers sustained responses and results in the persistence of many cancers despite therapy.

These drawbacks are driving the exploration of metabolic therapeutic strategies. One example among them is the use of a ketogenic diet (KD), a high-fat and low-carbohydrate eating plan that increases circulating ketone bodies such as β-hydroxybutyrate while reducing glucose availability [2, 3]. Rather than serving as a standalone cancer therapy, the KD has been investigated as a potential sensitization strategy that may target multiple biological pathways, including tumor metabolism, immune function, oxidative stress, and signaling pathways [3].

### Metabolic and immune reprogramming under ketogenic conditions

Under KD, cancer cells reduce glycolytic flux due to decreased glucose availability: tumour lactate production falls and the NADH/NAD⁺ ratio shifts, relieving extracellular acidosis and HIF-1α-driven immunosuppression in the microenvironment [1, 2]. The resulting rise in local NAD⁺ and fall in lactate partially rebalances redox signalling and helps preserve T-cell effector function. Simultaneously, elevated ketone bodies provide an alternative fuel for oxidative immune cells: CD8⁺ T-cells and other effectors can oxidise β-hydroxybutyrate, boosting mitochondrial membrane potential, TCA-cycle flux and acetyl-CoA production, which sustains their proliferation and function rather than allowing terminal exhaustion [1]. Importantly, β-hydroxybutyrate is itself a signalling metabolite and epigenetic regulator: it can engage with immune cell receptors (e.g. HCAR2) to dampen NF-κB/NLRP3-mediated inflammation, and it acts intracellularly as a class I histone deacetylase inhibitor, while altering transcriptional networks in immune and tumour cells toward stress resistance or anti-tumour states [2]. KD also remodels the gut microbiota by increasing Akkermansia and other species that produce short chain fatty acids, so that levels of beneficial short-chain fatty acids (like butyrate and propionate) rise. These microbiome-derived metabolites further reinforce the immunometabolic crosstalk, for instance by promoting differentiation of T-cells, enhancing the integrity of epithelial barrier and inhibiting histone deacetylases to support sustained gene-regulation changes [2, 3].

### Tumour metabolic stress and therapy sensitisation via a systems-level state

At the tumour-cell level, KD may enhance cancer treatment by stressing tumour metabolism: lowering insulin/IGF1 and mTORC1 signalling synergises with PI3K/Akt-targeted inhibitors, while glucose restriction depletes nucleotide and NADPH pools, making cancers cells more vulnerable to oxidative stress and DNA damage during chemo- or radiotherapy [1, 3]. For example, ketone-induced oxidative stress in tumour mitochondria can increase radiation related free-radical damage, while KD’s insulin suppression can strengthen the effects of targeted inhibitors of growth pathways such as PI3K and mTOR [3]. In this integrated biological state, changes in nutrient availability and cellular signalling influence immunity, metabolism, the microbiota, and epigenetic regulation all of which results in enhancing the effects of chemotherapy, radiotherapy, targeted therapy, and immunotherapy [1, 4].

During the course of this review, the ketogenic diet (KD) will be understood as a particular dietary approach involving significant carbohydrate restriction coupled with elevated fat intake sufficient to induce nutritional ketosis. The KD will be separated from long-term fasting, fasting mimicking diet (FMD), glucose restriction and exogenous ketone administration as interventions which can mimic aspects of the metabolism associated with ketosis, but which do not qualify as KDs themselves. The use of pharmacological interventions targeting metabolic processes such as metformin and mTOR-targeting compounds will similarly be regarded as independent treatments for consideration in potential combinations, and not components of the KD itself.

## The metabolic foundation of ketogenic therapy

### Glucose restriction and tumour metabolic vulnerability

Many types of cancers exhibit the Warburg effect, which is characterized by a reliance on aerobic glycolysis to produce energy and build new compounds [3, 5]. Such reliance is being used by the KD, as it provides a strong restriction in the total amount of systemic glucose. Due to the lack of carbohydrates in the diet, tumor cells, which usually upregulate glycolysis, experience metabolic stress. Indeed, the implementation of ketogenic feeding lowers the concentration of glucose in the bloodstream and raises the concentration of ketone bodies, which cannot be used effectively to create ATP by many tumor cells [3]. KD also leads to the downregulation of key enzymes responsible for glycolysis (e.g., pyruvate kinase M2, PKM2), thus lowering glucose absorption and lactate creation [5].

Lowering glycolytic flow makes tumor cells lose metabolic flexibility and ability to switch to other types of metabolism, thus forcing tumor cells into oxidative processes, which they cannot handle. It also disrupts redox balance and restricts the flow of glycolytic intermediates, including intermediates of the pentose phosphate pathway that can be necessary for nucleotide synthesis and creation of antioxidants (NADPH/ glutathione) [3, 6].

However, importantly, the metabolic limitation is not likely to affect all cells to the same degree. Selective effect on tumors as opposed to immune cells results from the difference in metabolic flexibility and not from an inability of the tumor cells to use ketones as a source of energy. Normal and immune cells are able to shift from glucose to fatty acid and ketone bodies oxidation depending on their availability, whereas specific subpopulations of tumor cells show high glycolytic dependence, malfunctioning mitochondria or deficiency of enzymes necessary for ketones metabolism. In this way, systemic ketosis may result in relative metabolic disadvantage for glycolytic tumor cells compared to normal and immune cells that have the metabolic potential.

Increased oxidative stress may be experienced by cancer cells in the case of glycolysis impairment. The cancer cells characterized by defective or stressful mitochondrial respiration become more dependent on oxidative metabolism, which may result in higher ROS production. In fact, KD was found to increase mitochondrial ROS in tumors [5, 7]. Such oxidative stress can be especially damaging for cancer cells already exposed to hypoxia, oncogenic signaling or replication stress.

In addition, KD decreases the activity of the insulin/IGF-1 pathway and stimulates energy sensors like AMPK that inhibit growth pathways such as PI3K/Akt/mTOR signaling. Thus, this combination of actions results in the creation of a metabolically restrictive microenvironment that features low glucose levels, decreased glucose metabolism rate, dysregulated redox homeostasis, and enhanced oxidative stress. It is important to emphasize that KD-induced starvation of cancer cells is not the primary mechanism behind the therapy; instead, it is a metabolic inequality because cancer cells cannot metabolically adapt to glucose shortage, while normal and immune cells can switch to oxidative metabolism.

### Ketone bodies as signalling molecules and immune metabolic fuel

The circulating ketone bodies consist mainly of β-hydroxybutyrate (BHB) and acetoacetate (AcAc); however, acetone is produced via the spontaneous decarboxylation of AcAc and is of little importance as an oxidative fuel. During prolonged nutritional ketosis, BHB becomes the main circulating ketone body and is not only an energy substrate but also a signal molecule. AcAc can also be transported to peripheral tissues and oxidized to acetyl-CoA; however, depending on the redox status of the cell, the amount of AcAc is variable relative to BHB. As for acetone, it is mainly exhaled and is of little significance as an oxidative fuel. In conclusion, the metabolic effects of ketogenic diets (KD) are mainly due to BHB and AcAc, while acetone should be considered more as a by-product of ketogenesis rather than a significant energy substrate.

Moreover, immune cells can oxidize ketone bodies for their cellular functions. Recent research demonstrated that activated CD8 + T cells could use ketones as an oxidative fuel [8]. Ketone oxidation leads to the production of acetyl-CoA, which enters the citric acid cycle, participating in the histone acetylation process. This shows how cellular metabolism influences gene expression and cell phenotype. For example, the absence of enzymes responsible for ketone oxidation impairs T-cell proliferation and its anti-tumor activity; thus, the metabolic processes of ketone bodies participate in anti-cancer immunity [8].

However, tumor utilization of ketones is heterogeneous and is based on the metabolic profile of each type of cancer. Tumors with low levels of ketolysis enzymes such as succinyl-CoA:3-ketoacid CoA transferase (SCOT/OXCT1) lack the ability to use BHB and AcAc as oxidative fuels, which makes them rely on glycolytic substrates. Some tumors have functional ketolytic pathway and are adapted to ketosis, and this poses a limit to the metabolic selectivity hypothesis. Thus, the advantages of KD are mostly seen in tumors which are highly glucose-dependent but cannot produce enough metabolic activity to compensate with ketone oxidation.

In addition, BHB may have signaling properties irrespective of its function as a fuel. It may act on hydroxycarboxylic acid receptor 2 (HCAR2) and regulate signaling pathways involving NF-κB and NLRP3 inflammasome [6, 9]. Likewise, AcAc may affect the cellular signaling and metabolic processes; however, compared to BHB, its role is much less described. At the moment, there is a lack of experimental evidence that AcAc alone, irrespective of its function as an oxidative substrate, is involved in cancer therapy sensitization. Moreover, BHB serves as an endogenous inhibitor of class I histone deacetylases that causes changes in chromatin acetylation and transcription [6, 9]. Thus, the effects of ketone-body signaling connect the metabolism in the organism and transcriptional regulation in the cell. However, the outcomes of the signaling by ketones vary greatly depending on the circumstances and cannot be viewed as anti-tumor effects for all cancer types.

On the other hand, acetyl-CoA formed during the process of ketolysis can be used for histone acetylation and regulation of transcription in immune cells [8]. Therefore, in ketosis, the same metabolic conditions that limit the glucose supply to tumor cells dependent on glycolysis may provide immune cells with alternative oxidative substrates and signals.

This dichotomy between tumours and immune cells is critical in the context of the hypothesized mechanism of KD. Still, it is important to recognize the relative nature of the selectivity between the two types of cells. There is metabolic heterogeneity amongst tumour cells, and some tumour cells may oxidize ketone bodies or develop adaptations to metabolic restrictions; at the same time, there is considerable variation in the response to ketosis in immune cells based on their phenotypic characteristics and physiological conditions. The potential efficacy of KD will, therefore, depend on the metabolic properties of the particular tumour and of the host immune compartment. Restriction of the tumour cells’ ability to utilize glucose and possible promotion of alternative fuel sources in immune cells through KD may provide an environment in which susceptible tumours would be compromised while the ability to eliminate tumours via anti-tumour immune response is maintained [5–9].

It is worth considering that ketone bodies belong to the class of lipids, and reports on the ability of lipid-lowering drugs to increase the efficacy of immune checkpoint inhibitors should also be taken into account. These data do not contradict the rationale behind KD, since they address different metabolic nodes: lipid-lowering drugs primarily reduce circulating cholesterol and influence cholesterol-dependent immune cell and myeloid cell signalling; whereas KD leads to an increase in circulating ketone bodies and fatty-acid oxidation substrates without necessarily leading to an increase in serum cholesterol concentration. Therefore, due to the difference in the mechanisms of action, it should not be assumed that effects of these interventions can simply add up.

## Immune reprogramming under ketogenic conditions

### T cell metabolic fitness

Beyond creating metabolic stress on tumour cells, the ketogenic diet (KD) may also reshape the immune environment into a more therapy-responsive state [10, 11]. New evidence suggests that ketosis does not simply “activate immunity” in a generalized sense; rather, its effects on immune cells are cell-type- and context-dependent, with some studies indicating enhanced persistence, functionality, and anti-tumour capacity of cytotoxic lymphocytes alongside potential weakening of immunosuppressive networks within the tumour microenvironment (TME) [10, 12]. Because of the highly regulated mechanism of immune cells, the nutrient and signaling changes induced by KD including reduced glucose availability, increased levels of ketone bodies, lower insulin signaling, and altered inflammatory mediators can directly affect the balance between immune activation and exhaustion [10, 12]. This rewiring of immune metabolism may be particularly important in cancer, where chronic antigen exposure, hypoxia, lactate accumulation, and nutrient competition progressively impair effective anti-tumour immunity [13, 14].

One of the most important effects of KD appears to include the enhancement of CD8 + cytotoxic T-cell metabolic fitness [12, 15]. Under tumour microenvironment, the activation of T cells poses competition with rapidly dividing tumour cells for glucose. Because of the high glycolytic rate of many tumours, due to the Warburg effect, glucose shortage can result in severe impairment of T cells’ effector functions [9, 13]. This raises a seeming paradox about KD because the limitation of glucose can affect not only the proliferation of tumour cells but also T cells that require glucose for their proper functioning. Nevertheless, the alleged selectivity of KD is not based on the sole restriction of glucose. Instead, it can be related to different flexibility of metabolism of these two types of cells. In the absence of glucose T cells undergo mitochondrial dysfunction, decreased cytokine production, compromised proliferative capacity along with exhaustion [13]. Some immune cell populations may reduce their dependency on glycolysis under KD and enhance oxidative metabolism (OM), FAO, and utilization of ketone body-derived metabolic fuels [12, 15, 16].

Ketone bodies like BHB, apart from being alternative metabolic substrates, also function as signaling metabolites that affect stress response and mitochondria function in a type and/or metabolic state-specific manner in immune cells [16, 17]. Consequently, KD offers an alternative metabolic milieu where T cells having the metabolic flexibility to generate energy via OXPHOS and other oxidative mechanisms even under low-glucose conditions can be sustained [12, 16].

Furthermore, the optimal mitochondrial function has been closely linked to persistence of T cells and long-term effectiveness against tumor cells [12, 13, 15]. Exhausted T cells usually have fragmented mitochondria and impaired energy production which leads to a gradual decline in cytotoxic function. Mitochondrial biogenesis, redox balance stability, and reactive oxygen species (ROS) damage are better supported using a ketogenic metabolism which may work against that process [12, 16].

Ultimately, after being active for long periods of time through immune activation, CD8 + T cells could potentially have higher levels of proliferation and continue to function longer than they would normally have been able to do so [15]. Experimental studies suggest that ketogenic or fasting-mimicking conditions may reduce the expression of exhaustion-associated markers such as PD-1, TIM-3, and LAG-3 in some experimental settings, suggesting that metabolic interventions may partially alleviate features of T-cell dysfunction [10, 12]. This is mechanistically important because T-cell exhaustion represents one of the major barriers limiting durable responses to modern immunotherapies [10, 13].

### Tumor microenvironment modulation

In parallel, KD may reprogram the tumour microenvironment in a way that reduces inflammation driven immune suppression [10, 14]. Tumours often establish a long lasting inflammatory environment that is also immune suppressive, characterized by hypoxia, lactate accumulation, acidic pH, suppressive cytokines, and dysfunctional stromal signaling [9, 14]. Aerobic glycolysis generates high amounts of lactate that inhibit the activity of cytotoxic T cells, impede the ability of dendritic cells to present antigens, and encourage the proliferation of regulatory immune populations [9]. By reducing glycolytic flux and lactate production, ketone bodies may reverse the metabolic suppression associated with lactate and enhance the functionality of immune cells in tumors [10, 11]. Decreased systemic glucose and insulin signaling may also further down regulate the activation of pro-tumorigenic pathways such as PI3K/Akt/mTOR, indirectly affecting inflammatory cytokine networks and tumour associated stromal interactions.

Ketone bodies themselves may also have anti-inflammatory signaling properties.β-hydroxybutyrate has been shown to inhibit activation of the NLRP3 inflammasome which is a key mediator of chronic inflammatory signalling, contributes in progression of tumour and dysregulation of immune system [18]. Through this mechanism, ketosis may down regulate the synthesis of inflammatory cytokines such as IL-1β and IL-18, and transform immune responses from chronic pathological inflammation to more effective cytotoxic immunity. This does not necessarily indicate general immune suppression, but rather KD may selectively reduce dysfunctional inflammation while maintaining or increasing anti-tumour immune response [10]. Such a shift might be important, since chronic inflammation within tumours often promotes immune exhaustion rather than productive immune monitoring [14].

Immunosuppressive and regulatory T cell populations are affected by ketosis in the tumor microenvironment. Tumor associated macrophages (TAM) support angiogenesis by developing an m2 like phenotype, remodeling the extracellular matrix, and assists in immune evasion of the tumor [19]. Preclinical studies have indicated that KD might re-polarise macrophages towards a more inflammatory and antitumour M1-like phenotype [10, 19, 20]. This may result in an increased antigen presentation and eventually in an increased recruitment of effector lymphocytes and a decreased immunosuppression of the tumour.KD could also affect Treg proliferation, survival and suppressive capabilities by changing the local nutrient composition and cytokine signaling environment in the TME in a way favorable to these processes. However, contrary to the generally positive effects of the CD8⁺ T cell metabolic fitness, the impact of ketosis on the Tregs is unlikely to be uniform and is likely to vary depending on tumor type, location, disease stage, and specifics of a particular diet or metabolic therapy [10]. In addition, KD cannot be viewed as an inherently immunostimulatory diet; rather, its potential to tip the immune balance towards effective tumour clearance is probably dependent on the response of different populations.

A possible reason for this is the difference in metabolic needs and adaptability of the CD8⁺ T cells and Tregs in their activated state: while activated effector CD8⁺ T cells may have a benefit from the presence of oxidative substrates and increased mitochondrial fitness, Treg activity and proliferation are known to be affected significantly by local nutrient composition and cytokine signaling. This means that the changes in the levels of glucose, ketone bodies, fatty acids, insulin/IGF-1 signaling and other inflammatory factors could affect these populations differently rather than create a single effect across all T cell populations.

In particular, Treg suppressive capacity and stability in many cases require fatty-acid oxidation and oxidative phosphorylation and Treg proliferation and maintenance are known to be affected by the local nutrient composition in the TME; in other words, ketosis can change the nutrient balance competition between Tregs and effector CD8 + T cells rather than provide an advantage to one population over another.

### Synergy with immunotherapy

These immunometabolic effects have led to increasing interest in combining KD with modern immunotherapies. In particular, immune checkpoint inhibitors (ICIs), such as anti-PD-1, anti-PD-L1 and anti-CTLA-4, work through the reactivation of exhausted T cells. Many patients, however, do not respond because severe metabolic dysfunction in the tumour microenvironment prevents T-cell recovery even after checkpoint blockade.KD may sensitise tumours to checkpoint inhibition by restoring mitochondrial fitness, lowering exhaustion signalling and decreasing metabolic competition.

Preclinical models have shown that combining ketogenic interventions with PD-1 blockade improves responses, suggesting that metabolic therapy could convert immunologically “cold” tumours into more therapy-responsive states [21]. There may also be potential synergy with adoptive cellular therapies such as CAR-t therapy. The metabolically hostile tumour microenvironment, characterised by hypoxia, nutrient deprivation and chronic antigen stimulation, presents one of the major challenges limiting CAR-T efficacy in solid tumours. Engineered T-cell survival is compromised in this environment .Ketogenic states could improve metabolic flexibility and resistance of transferred T-cells by enhancing oxidative metabolism and increasing mitochondrial robustness [12, 16]. Enhanced mitochondria promote memory like T-cell states, which are especially important for sustaining prolonged CAR-T therapy responses [12, 15].

Additionally, since the level of inflammation suppression in the tumor environment will decrease, it would enable increased penetration of adopted T-cells and their sustained cytotoxicity. As in the case of other kinds of adoptive cell therapy approaches including tumour infiltrating lymphocyte therapy and engineered t-cell receptor therapy, there can be an impact on the efficacy of the method through the application of a metabolic intervention aimed at enhancing the function of the immune system [10, 12]. As the efficacy of the above methods is largely contingent upon the survival and functioning of adopted cells, any enhancement of immune function will increase the efficacy of the method.

Overall, the immunologic effects of KD appear to be much broader than simply depriving the body of nutrients. KD may contribute to reprogramming immunity towards a more effective anti-tumour state by improving T-cell metabolic fitness, decreasing immune exhaustion, modulating inflammatory signalling and weakening suppressive tumour microenvironment networks, all at once [10, 12, 14]. This systems- level immune reorientation may be why ketogenic interventions show the greatest promise when used alongside existing therapies, rather than as stand-alone treatment [10, 11]. Here, KD could be a metabolic framework that can synchronise tumour stress and immune activation to enhance the therapeutic responsiveness of cancer to modern immunologic interventions [10]. 

## Microbiome–epigenetic regulatory axis

### KD-induced microbiome remodeling

However, ketogenic diet (KD) can affect the microbiome composition and metabolic and immune properties which can play a role in influencing cancer progression and responsiveness to treatments. By significantly limiting carbohydrates in the diet while promoting fats, KD changes the nutrients available for intestinal microbes, which in turn can change the composition and metabolism of microbial communities. Several studies have shown that KD can affect the relative abundance of taxa involved in inflammatory signaling and lipid metabolism, but the extent and nature of changes depend on diet composition, diet duration, host factors, and diseases context [22–24]. However, the mere change in microbiome composition does not mean anything, since its potential effect should be determined by the alteration of microbial metabolites, permeability of intestinal barrier, and immune response.

In addition, the changes in microbial composition might affect immune regulation and inflammatory signaling through microbial metabolites and the state of the intestinal barrier. The gut microbiome controls the host immunity through microbial metabolites, products and interaction with intestinal epithelial and immune cells. For example, microbiome-derived metabolites can affect immune-cell functions and differentiation, such as T-cells response and macrophages polarization. Such mechanism provides a link between diet-induced changes and systemic immunity. Reduction of inflammatory mediators, like IL-6, TNF-α, and cytokines responsible for chronic tumor-associated inflammation has been observed in some studies under the action of ketogenic diets [18, 25]. At the same time, alterations in microbial metabolism can affect anti-inflammatory signaling pathways [23].

There is new evidence about the possibility of influencing anti-tumor immunity and therapy response by means of the microbiome. The composition of certain taxa of bacteria and their metabolites can be responsible for such processes as antigen presentation, T cell activation, cytokine production, and the state of functional myeloid cells [25]. All these can affect the presence of either permissive or suppressive environment in the tumor. It means that there is a potential connection between the microbiome remodeling caused by KD and therapy sensitization: it is possible that the dietary intervention alters the production of certain microbial metabolites, affecting intestinal and systemic inflammation, metabolic state of immune cells, and activities of effector lymphocytes, thus influencing the response of such treatments that are based on the functionality of anti-tumor immunity, especially immune checkpoint inhibition. The connection between changes in microbiome associated with KD and improvement of response to cancer treatment is largely preclinical, and the connections between particular microbial changes and response to treatment are not proven to be causal. Additionally, microbial metabolites may have an influence on oxidative stress response and inflammatory signaling, providing further connections between microbiome metabolism and cellular stress responses used in tumor therapy [19].

Another important feature of the microbiome remodeling is intestinal permeability. The barrier is created by the intestinal epithelium, mucus layer, antimicrobial molecules, and tight-junction complexes regulating the passage of substances in the intestinal lumen into the bloodstream. It is possible that the disruption of this barrier will result in increased translocation of microbial components (for example, lipopolysaccharides and other molecules recognized by innate immunity) to the systemic circulation, thus causing systemic inflammatory signaling. KD can influence the integrity of the intestinal barrier indirectly through its influence on microbiota composition and metabolite production, which affects epithelial cells’ metabolism, mucus production, and regulation of tight junctions. In this way, the diet decreases the risk of increased intestinal permeability due to reduced inflammation and epithelial dysfunction. One of the mechanisms of action is the following: short-chain fatty acids (especially butyrate) produced by KD-associated bacteria serve as a preferred source of energy for colonocytes and can help to maintain the expression of tight junctions. However, it is unknown how consistent and influential in human experiments this effect is.

### Epigenetic effects of ketone bodies

Ketone bodies not only serve as a source of energy but they are also important signaling molecules. The important ketone body, β-hydroxybutyrate (BHB), has the ability of gene regulation. BHB is an inhibitor of histone deacetylases (HDACs). HDACs are enzymes that remove acetyl groups from histone proteins and promote chromatin condensation. HDAC inhibition leads to a chromatin that is more favourable to transcription [26].

By inhibiting HDACs, BHB can regulate the expression of genes involved in oxidative stress resistance, inflammation, cellular differentiation, and immune regulation [26, 27]. Increased histone acetylation may cause transcription of antioxidant pathways and suppress pro-inflammatory signaling pathways that are associated with tumors. Epigenetic modulation that is induced by ketone bodies may cause altered gene expression in the PI3K–AKT–mTOR pathway and inflammatory mediators [28]. This highlights the role of ketone bodies as important modulatory molecules and not merely a source of energy.

Another key aspect is tumor plasticity. Tumor cells adapt to a great extent in order to survive hypoxia, nutrient deprivation, and therapeutic stress by regulating gene expression. KD may prevent this by altering gene expression and as a result make tumor cells more responsive and sensitive to therapy [29].

Importantly, these epigenetic changes are not limited to tumor cells only. Immune cells that are present in the tumor microenvironment can also show response to ketosis. Consequently, KD may be involved in coordinated regulation of both tumor and immune cells, further validating its proposed role as a systemic sensitization strategy in oncology [30].

### Integrated immune–epigenetic crosstalk

The interaction of the microbiome, immune system regulation, and the epigenetics of the ketogenic diet’s influence on cancer therapy is a fundamental and pivotal mechanism by which the KD has an effect upon cancer therapies. The KD is initially thought to only offer an alteration to glucose metabolism by KD, however, it also produces epigenetic changes that can lead to changes in metabolism and ultimately alter systemic signaling pathways [19, 26].

In terms of the effects of the KD on antitumor immune responses, it appears to enhance antitumor immune responses and decrease immunosuppression within the TME (tumor microenvironment), via the reduction of systemic inflammation and the overall change in immune signaling; ketone bodies produced during the KD are also thought to cause epigenetic changes that would increase the ability of T-cells to respond, plus regulate genes that regulate how the immune system will respond to tumors, and tumors’ capacity to survive [30, 31].

Furthermore, the KD may reduce the ability of tumors to adapt and modify themselves, thus reducing their ability to overcome therapies. In addition, the microbiome likely plays a key role in these mechanisms by providing metabolites that influence immune and epigenetic signaling pathways, thereby increasing the total amount of systemic reprogramming of the tumor biology, plus may impact multiple systems and pathways instead of a single pathway [23, 25].

Many of the references related to this concept have been preclinical studies; however, this relationship between the microbiome and epigenetics is a very solid starting point for examining the therapeutic effect of ketogenic diets on cancer patients.

## Ketogenic diet as a therapy sensitization platform

### Chemotherapy sensitization

The ketogenic diet (KD) has attracted interest as a potential therapy-sensitization strategy in oncology, particularly in preclinical studies. Rather than functioning primarily as a stand-alone anticancer treatment, KD has been investigated as a metabolic adjunct that may increase tumour vulnerability to conventional therapies by altering glucose availability, redox balance, and stress-response pathways. This may be achieved by altering tumor metabolism and making tumors more vulnerable to therapeutic stress. In chemotherapy, increasing the oxidative stress is one of the most important ways in which KD can increase the sensitivity of the tumor cells.

Tumor cells very often exist in a state that is very stressed due to the increased production of reactive oxygen species (ROS). There is also an increased dependence on glucose for survival. This effect is expected to be most relevant to tumors with substantial glycolytic dependence and limited metabolic flexibility, rather than representing a universal response across all cancers [29, 32].

In order to survive the toxic effects of chemotherapy, tumor cells often rely on glycolysis and other compensatory metabolic pathways. Tumor cells may be forced towards adapting mitochondrial oxidative metabolism due to decreased glucose by KD. Because some tumors have impaired mitochondrial function or limited ketone utilization, their ability to compensate for reduced glycolytic flux may be constrained under ketogenic conditions [19, 29]. As a result, there may be increased sensitivity of tumor cells to DNA damage and apoptosis.

In addition to this, growth-inducing pathways, such as insulin/IGF-1 and PI3K–AKT–mTOR signaling, may be inhibited by KD, further increasing its sensitivity to chemotherapy [33]. Anabolic activity and cellular growth may be limited by lower insulin signaling. As normal tissues possess greater flexibility in their metabolism, experimental studies have suggested that ketogenic metabolic conditions may increase oxidative stress selectively in tumor cells [34].

One other key aspect is the influence that KD has over systemic inflammation and the tumor microenvironment. Chronic inflammation results in the promotion of survival signaling and suppression of effective immune responses. Through reduction of inflammatory mediators and metabolic reprogramming, KD may create a microenvironment more favorable to chemotherapy responsiveness [18, 35]. Furthermore, ketogenic interventions may improve patient tolerance to therapy by stabilizing blood glucose fluctuations and reducing systemic metabolic disturbances.

Although many findings remain preclinical, available evidence supports further investigation of KD as a metabolic adjuvant capable of enhancing chemotherapy-induced tumour stress. However, whether KD can improve treatment efficacy without increasing clinically relevant toxicity remains uncertain and requires evaluation in adequately designed clinical studies. This makes KD an attractive non-pharmacological strategy for combination therapy in oncology.

### Targeted therapy synergy

In addition to chemotherapy, KD has been investigated in combination with several targeted metabolic and signaling therapies. However, the evidence is substantially less mature and more heterogeneous than the evidence base for immunotherapy, and most proposed combinations remain supported primarily by mechanistic or preclinical studies. These effects are mainly related to the metabolic and signaling pathways that regulate the growth and survival of the tumor cells.The PI3K–AKT–mTOR pathway is one of the most important pathways that are influenced by both KD and targeted agents. This pathway plays a key role in cellular proliferation, metabolism, and resistance mechanisms. The activity of mTOR inhibitors may be complemented by KD since it reduces insulin and glucose signaling. This results in further suppression of the anabolic signaling pathways [33, 36].

The best explanation of this strategy can be found in the correlation between KD-related impairment of the insulin/glycogen signaling pathways and pharmacological suppression of the growth-related pathways downstream. However, the data on the efficacy of such treatment is limited, and the scientific basis of this combination should not be confused with its therapeutic benefit [33, 36].

The use of KD with metformin is also of significant interest. Metformin suppresses hepatic gluconeogenesis and alters cellular energy metabolism, with effects involving mitochondrial complex I inhibition and AMPK-related signaling among other mechanisms [37]. Therefore, KD and metformin may produce even more metabolic stress on tumor cells by limiting glucose supply and impairing mitochondrial adaptation [37]. However, evidence for a clinically meaningful KD–metformin synergy in cancer remains preliminary and should be distinguished from the more established use of metformin as a metabolic modulator.

Another collaboration is between KD and isocitrate dehydrogenase (IDH) inhibitors. Tumors having mutated IDH show altered metabolic and epigenetic states. This may result in increased dependence on specific metabolic pathways. Because IDH mutant tumors exhibit specific metabolic and epigenetic vulnerabilities, the presence of ketogenic states might theoretically affect these vulnerabilities by means of metabolic and oxidative redox alterations. Unfortunately, experimental proof confirming an increased efficiency of IDH inhibitors due to ketogenic states is insufficient [38].

Long term efficacy of therapies is affected by certain adaptive resistance pathways. KD may also affect these pathways by limiting nutrient availability, which can reduce the tumor’s ability to activate alternative survival mechanisms [19]. Its benefits are not only direct on cancer cells but also indirect through effects on immune response, inflammation, and oxidative stress. On balance, the use of combination therapy is a biologically rational follow-on from the system-based approach; however, the degree of evidence supporting each particular combination is highly variable. In its current state, such strategies should mainly be viewed as being preclinical or experimental.

### Radiotherapy and oxidative stress

Radiotherapy suppresses tumor growth by generation of ROS and induction of DNA damage. As cancer cells operate near the threshold of their oxidative stress, even a slight increase in it will overcome their defences. Ketogenic diets may enhance this vulnerability by altering tumor metabolism and reducing glucose-dependent antioxidant capacity [34, 39].

Under ketogenic conditions, there may be increased oxidative stress in tumor cells due to impaired glycolysis and NADPH generation. Pentose phosphate pathway which is an important source of reducing equivalents used for antioxidant defense may be inhibited due to inadequate glucose supply. As a result, tumor cells may become more sensitive to oxidative stress induced by radiation [13].

At the same time, normal healthy tissues possess a greater degree of adaptation. They are also much better at utilizing ketone bodies efficiently for energy production. This metabolic flexibility may result in reduction of oxidative stress in normal cells compared to metabolically weak tumor cells; however, selectivity to protect normal cells has not been proven clinically [19].

Experimental studies have demonstrated that in pre-clinical cancer models, ketogenic interventions may increase radiation sensitivity and delay tumor growth [28, 39]. Some proposed mechanisms for the increased sensitivity to radiotherapy are increased vulnerability due to ROS, impaired DNA repair, and reduced metabolic compensation. Additionally, the decrease in inflammation and vascular abnormalities by KD may further improve treatment responses within the tumor microenvironment.

While there is limited clinical data, pre-clinical research shows promise that KD might increase oxidative stress and radiosensitivity in metabolically sensitive tumors. However, whether this leads to better tumor control and lower normal tissue damage in patients is not known for certain. This approach emphasizes the broader concept of metabolic therapy sensitization in the treatment of cancer.

### Immunotherapy enhancement

Out of all the various forms of therapy that have been discussed, immunotherapy appears to be one of those with a very strong rationale for combining with KD from a mechanistic point of view despite lacking clinical evidence. One of the crucial factors influencing T-cell viability and activity is immune cell metabolism, and recent developments in immunotherapy have made this area more relevant. Active T cells that are capable of surviving in the nutrient deficient tumor microenvironment are key to effective immune response against tumor cells. However, T cell function is frequently impaired by tumors because they create conditions of glucose competition, lactate accumulation, and chronic inflammation [31].

Ketogenic diets may improve immunotherapy responsiveness by supporting immune cell metabolism and reducing tumor-associated immunosuppression. Ketone bodies such as β-hydroxybutyrate (BHB) can influence inflammatory signaling and immune regulation through epigenetic and metabolic mechanisms [26, 30].Moreover, decreased glucose availability might be detrimental to glucose-dependent cancer cells through glycolysis, but immune cells that are metabolically flexible might be able to make up for this somewhat with oxidizable substrates; on the other hand, too much glucose limitation might be damaging to the functionality of immune cells.

KD may also influence the gut microbiome in ways that enhance responsiveness to immune checkpoint inhibitors. Certain bacterial populations are associated with improved T-cell activation and better outcomes during anti-PD-1 therapy [25]. Gut microbiota modifications associated with KD might be able to affect tumour immunotherapy through metabolites produced by the microbiota, the status of the intestinal barrier and further inflammatory signaling pathways; however, the effect of these modifications on ICI therapy is not yet clear.

Another important mechanism involves reduction of chronic inflammatory signaling pathways that contribute to immune suppression within the tumor microenvironment. Lower levels of inflammatory cytokines and metabolic stress may improve T-cell persistence and cytotoxic activity [25, 35]. Furthermore, ketone-mediated epigenetic regulation may alter transcriptional programs involved in immune activation and tumor immune evasion [26].

Together, these studies indicate that KD can serve as an immunometabolic therapy intervention that is able to alter the metabolic and inflammatory environment that affects the immune treatment outcome. However, the majority of studies at the moment are preclinical, and further clinical trials are necessary to clarify if the benefits in mechanisms have any effect on the outcome and response to immunotherapy in terms of higher response rates, progression-free survival, and overall survival. As regards other combination therapies that were discussed above in the current section, KD combined with immunotherapy is relatively the most developed field for translational research.

Table 1 summarizes the proposed mechanisms, evidence base, and current translational status of KD across major anticancer treatment modalities.

**Table 1:** Ketogenic diet as a therapy-sensitization strategy

Therapy	Proposed Mechanism	Evidence Base	Current Status
**Chemotherapy**	↑ Oxidative stress; ↓ glucose/redox support	Mainly preclinical; limited clinical studies	Investigational
**Targeted therapy**	↓Insulin/PI3K–AKT–mTOR signaling; metabolic stress	Mainly preclinical	Hypothesis-generating
**Metformin**	Complementary metabolic and mitochondrial stress	Preclinical/mechanistic	Investigational
**IDH Inhibitors**	Metabolic, redox, and epigenetic vulnerability	Limited preclinical evidence	Hypothesis-generating
**Radiotherapy**	↑ ROS; ↓ antioxidant capacity	Mainly preclinical; limited clinical evidence	Investigational
**Immune checkpoint inhibitors**	↑ T-cell fitness; ↓ metabolic competition; microbiome effects	Preclinical; emerging clinical evidence	Investigational
**CAR-T / adoptive cell therapy**	↑ Mitochondrial fitness and T-cell persistence	Mainly preclinical	Experimental

## Systems-level integration model

### Unified immunometabolic sensitization framework

The value of using the ketogenic diet as a means of therapy sensitization should not be viewed as based on one or several metabolic effects, but rather, the complex interplay of tumor metabolism, immunological cell functions, the gut microbiome, and epigenetics. This complex can be represented by multiple layers, rather than separate mechanisms. Ketogenic diet-related glucose restriction and the process of nutritional ketosis change glucose and insulin levels in blood and the level of available ketones, which can produce metabolic pressure for the cells. The metabolic pressure may be higher for tumors with a high glycolysis rate and low metabolic plasticity [29, 33].

Metabolic effects of KD can affect the immune compartment at the same time. The reduction of the tumor’s glycolysis rate and changes in the competition for nutrients in tumor microenvironment can affect the metabolic status of the infiltrating immune cells, while BHB and fatty acid oxidation can be used as alternative sources of oxidative substrate in metabolically plastic immune cell populations [23, 31]. All these effects can lead to improving mitochondrial fitness and functioning of some cytotoxic T-cells, but the effects of ketosis on the immune system will depend on the type of cells, their activation and tumor microenvironment. In other words, the selective effects of the KD are relative; the same reduction of glucose availability that affects the high glycolytic tumor cells can harm glucose-dependent immune cells.

Gut microbiome is yet another link between nutritional metabolism and systemic immunity. Changes in nutrient availability resulting from KD can change the composition and activity of microbes, which can result in changed production of metabolites by these microorganisms, as well as inflammatory mediators downstream from them [22, 25]. Alterations of the microbiome can also impact the state of the intestinal barrier and transfer of the products of the metabolism of the microbes to the circulation, thus affecting the systemic immune tone. The microbiome metabolites could also engage in interaction with immune and epithelial cells, giving yet more possible pathways of integration between diet, inflammatory signaling and the tumor microenvironment. Importantly, direct proof of an effect of changes in microbiome due to KD on increased efficacy of cancer therapy remains preclinical to date.

Another pathway linking nutritional metabolites to persistent alteration of cellular state would involve epigenetic regulation. Metabolites produced or altered during ketosis, such as BHB and acetyl-CoA, can influence chromatin and its modification [18, 26, 27]. The former compound was reported to be able to affect histone deacetylases in experimental settings, whereas the latter metabolite can affect histone acetylation. Yet, the extent to which these processes happen in physiological doses in the setting of KD in humans remains unknown. However, these interactions can nevertheless influence oxidative-stress responses, immune-cell function and tumor cell adaptation.

Thus, the general idea of the cascade is as follows: KD leads to changes in systemic nutrient and hormonal signal → changes in metabolic stress and metabolic conditions in immune cells → changes in microbiome and production of its metabolites → changes in inflammatory signaling in intestine and in the system → influence of metabolic and epigenetic signals on cellular adaptation and immune-cell functions → the combination of all these factors might render certain tumors more vulnerable to anti-cancer therapy. The strength and nature of each step will most likely depend on metabolic phenotype of the tumor, immune-cell composition, properties of the microbiome, treatment modality and specific ketogenic or metabolic intervention employed.

The framework proposed also accounts for the reason why KD has much more potential as an adjuvant than as a mono-therapy against cancer. Chemotherapy and radiation treatments cause DNA and oxidative stress, targeted treatments block specific survival pathways in tumor cells and immunotherapy requires functional anti-tumor immune response. Thus, by influencing multiple targets at once, KD can improve therapeutic outcome.

However, these interactions remain mechanistically and clinically heterogeneous, and evidence for improved treatment outcomes is substantially stronger in some therapeutic settings than others. The integrated pathways are summarized in Table 2, which links the major metabolic, immune, microbiome, and epigenetic effects of KD to their proposed contribution to therapy sensitization.

**Table 2:** Systems-level mechanistic framework of ketogenic diet in cancer therapy

Biological layer	Ketogenic diet–induced change	Downstream mechanistic effect	Cancer therapy relevance
**Metabolic Reprogramming**	↓ Glucose availability, ↓ insulin/IGF-1 signaling, ↑ β-hydroxybutyrate (BHB)	Tumor glycolytic restriction, ↓ NADPH/NADH imbalance, ↑ oxidative stress	Weakens tumor energy supply and increases susceptibility to therapy-induced stress
**Tumor Bioenergetics**	Reduced glycolysis, impaired PPP flux, altered mitochondrial stress	↓ nucleotide synthesis, ↓ antioxidant capacity, ↑ ROS accumulation	Enhances chemotherapy and radiotherapy sensitivity
**Immune Metabolic Rewiring**	Shift toward ketone/fatty acid oxidation in T cells	↑ CD8⁺ T-cell persistence, ↑ mitochondrial fitness, ↓ exhaustion (PD-1, TIM-3)	Improves anti-tumor immunity and immunotherapy response
**Inflammatory Signaling**	↓ Lactate accumulation, ↓ NF-κB and NLRP3 activation via BHB	Reduced immunosuppression and inflammatory tumor microenvironment	Reverses immune exhaustion and supports cytotoxic activity
**Microbiome Remodeling**	↑ SCFA-producing bacteria (e.g., butyrate producers), ↓ pro-inflammatory taxa	↑ SCFAs → T-cell differentiation + HDAC inhibition	Strengthens immune surveillance and systemic anti-tumor signaling
**Epigenetic Regulation**	BHB-mediated histone deacetylase (HDAC) inhibition	↑ Histone acetylation, reactivation of tumor suppressor/immune genes	Promotes transcriptional reprogramming toward anti-tumor states
**Therapy Sensitization Output**	Integrated metabolic + immune + epigenetic stress	“Therapy-sensitive state” in tumor biology	Enhances chemotherapy, radiotherapy, targeted therapy, immunotherapy

**Fig. 1:**
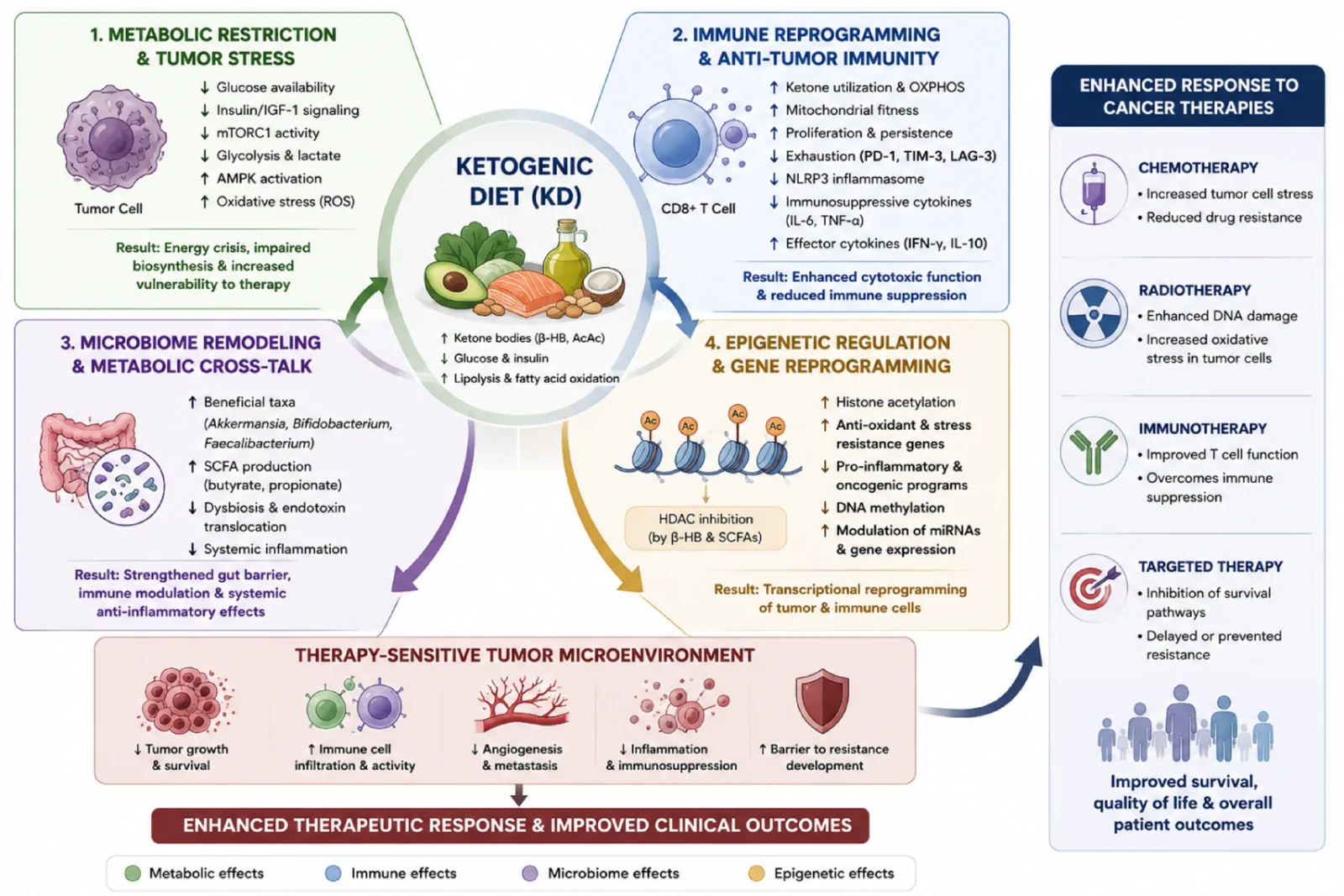
Systems-level immunometabolic model of the ketogenic diet in cancer therapy, integrating metabolic, immune, microbiome, and epigenetic effects to enhance therapy sensitivity

The systems-level immunometabolic model of KD is shown in Fig. 1.

## Clinical evidence and translational challenges

Despite the considerable mechanistic preclinical data favoring KD as an adjuvant treatment for cancer, clinical evidence is still lacking and not robust enough to support the integration of KD into routine oncology practice [7, 19]. The available clinical trials have focused more on feasibility, compliance, metabolic outcomes, and quality of life measures instead of any significant survival benefits of the treatment. It must also be noted that human results might differ from preclinical results due to different metabolism of the tumors, nutritional status, compliance with treatment, and success at obtaining nutritional ketosis. Therefore, KD should be currently regarded as a research adjuvant therapy.

### Current clinical evidence

In clinical studies in oncology, the study of KD still falls considerably behind preclinical research. In human investigations so far, KD was examined as a standalone nutritional therapy, and more often as an add-on therapy in patients with glioblastoma, breast cancer, and other gastrointestinal tumors [37, 40–44]. Various effects on metabolic parameters, body composition, QOL, treatment tolerance, disease-specific outcomes, and tumor regression have been shown depending on the studied aspects in different patient populations. Nevertheless, the above results were obtained in small prospective studies, pilot trials, retrospective observations, or monotherapy without control groups; therefore, it is difficult to define the clinical benefits that can be attributed to the KD.

The effect of KD, which has been consistently demonstrated in clinical trials, is the impact of the nutritional approach on systemic metabolism, such as reduction in the level of glucose and insulin signaling and an increase in ketone concentrations in the blood when nutritional ketosis occurs. The changes in metabolism represent the biological exposure to the therapy, but the above-mentioned indicators cannot serve as evidence of improvement in cancer control. Likewise, the decrease in body weight, levels of inflammation, fatigue or QOL indicators in individual studies need to be evaluated with care since they may depend on caloric intake, weight loss, additional therapy, nutritional state, and other factors.

However, evidence for antitumor efficacy is far more inconsistent. Some early reports suggest disease stabilization and potential improvement of response to existing treatment in the case of KD, while others do not provide proof of tumor control benefits. The variability in terms of the type of cancer, its stage, treatment approach, composition of diet, caloric intake, duration of treatment, and level of attained ketones makes comparative analysis challenging [37, 41]. Furthermore, the lack of randomized controlled study designs or the presence of a very small number of selected participants make distinguishing the real effect of the treatment from potential bias, the effect of other treatment or difference in nutritional support complicated.

Another limitation consists of the fact that nutritional ketosis itself is an exposure which cannot be considered uniform. Patients adhering to the apparently same ketogenic diet might achieve different blood levels of BHB depending on the factors listed above. For this reason, future studies should include objective measures of the compliance and achievement of ketosis rather than dietary composition alone.

Notably, one should also differentiate between the results of human studies and those gained in the experiments with fasting, fasting mimicking diets, glucose restriction or ketone supplementation. While these approaches allow to reproduce the metabolic characteristics of KD, they cannot substitute the diet per se in interpretation of clinical data.

Summarizing the information discussed above, one can conclude that currently the human evidence suggests the feasibility and activity of KD in some oncology population, but does not prove any benefits in terms of antitumor response, PFS or OS. Therefore, the most reasonable interpretation of the evidence is that KD is a promising but investigational metabolic intervention.

### Practical and biological limitations

Ketogenic diet with its potential benefits may improve cancer therapy but it still has some clinical and biological limitations. A major challenge is the maintenance of this diet for a prolonged period of time.The first challenge relates to adherence. It may be challenging to follow a strict diet and restrict intake of carbohydrates for long periods of time, especially when a patient suffers from anorexia, nausea, changes in taste sensation, fatigue, digestive complaints, or nutrient deficiencies caused by the treatment [19, 35]. The level of adherence can vary considerably between individuals as well as during the course of treatment and affect the level and duration of ketosis achieved. However, low adherence should not be seen only as a sign of treatment failure because of the challenges in keeping KD. This difference in adherence to the diet may significantly affect metabolic outcomes and this leads to inconsistent results in different studies [43].

Another major hurdle is overcoming the nutritional risks associated with ketogenic diet. Possible nutritional issues might be related to unintentional weight loss, improper nutrient consumption, possible GI symptoms, and the inability to maintain an appropriate energy and protein intake, especially considering KD therapy that is not properly controlled from a nutritional perspective [44]. These problems are likely to be especially significant in the case of advanced malignancy, cancer-related weight loss, or existing malnutrition, when nutritional complications might have a negative impact on the ability of a patient to tolerate treatment and its outcome. Thus, the use of KD in oncology requires personalized nutrition approach.

Another major challenge involves tumor heterogeneity and metabolic adaptability. Cancer is not metabolically uniform, and tumors may dynamically alter their nutrient utilization in response to environmental stress [9, 14, 32]. Some malignancies may compensate for glucose restriction by increasing fatty acid oxidation, glutamine metabolism or alternative bioenergetic pathways. This reduces the effectiveness of ketogenic diets in cancer therapy. This flexibility can also account for the differences seen in patients data in laboratory studies. This effect is dependent on an individual condition and is not the same for every patient.

Standardized ketogenic protocols are also lacking [10, 19]. Studies differ in carbohydrate thresholds, fat-to-carbohydrate ratios, total caloric intake, protein content, duration of intervention, methods used to monitor ketosis, and concurrent anticancer therapies. These differences make it difficult to determine whether discrepant clinical outcomes reflect differences in tumor biology or differences in the metabolic intervention itself. Future trials should therefore report dietary composition, caloric and protein intake, adherence, circulating BHB and glucose concentrations, and predefined metabolic targets whenever possible.

### Safety and caution

Although emerging evidence supports the possibility that KD may function as a multi-layered therapy sensitization strategy, it is critical to emphasize that KD is not a replacement for established cancer therapies. Current evidence does not support the use of ketogenic interventions as independent curative treatment for malignancy. Instead, the greatest potential value of KD may lie in its ability to create a biologic environment that enhances sensitivity to existing therapies through coordinated metabolic and immunologic modulation.

At present, many available studies remain exploratory and hypothesis-generating rather than definitive. Large, controlled prospective trials are still needed to determine which patients are most likely to benefit, identify optimal ketogenic protocols and evaluate long-term safety outcomes. Future studies should also incorporate biomarker-guided approaches capable of assessing metabolic flexibility, immune responsiveness and microbiome composition in order to better define ketogenic-sensitive tumor states [45].

Ultimately, successful clinical translation will require moving beyond the simplistic view of KD as merely a low-carbohydrate diet. Instead, KD should be investigated as a systems-level metabolic intervention capable of interacting with immune, epigenetic and therapeutic networks in a coordinated manner. Until stronger clinical evidence becomes available, ketogenic strategies should remain carefully supervised adjunctive approaches integrated alongside evidence-based oncologic therapies rather than substitutes for standard cancer treatment.

To place the mechanistic evidence in clinical context, Table 3 summarizes representative human studies evaluating KD as an adjunct to cancer therapy, emphasizing study design, clinical outcomes, limitations, and the current maturity of the evidence.

**Table 3:** Representative clinical evidence evaluating ketogenic diet as an adjunct to cancer therapy

Cancer type / setting	KD Intervention	Study Design/ Population	Concurrent treatment	Main clinical findings	Key limitations	Evidence maturity
**Glioblastoma / high-grade glioma**	Various KD protocols, including classic KD and modified Atkins approaches	Small prospective studies, pilot studies, case series and observational cohorts	Chemoradiotherapy, temozolomide or other standard therapy	Feasibility and achievement of ketosis have been demonstrated; some studies reported disease stabilization or signals of prolonged PFS/OS, but findings are inconsistent	Small samples, heterogeneous diets, lack of controls, historical comparators and variable adherence	Low / preliminary
**Glioblastoma**	Supervised 3:1 KD for 16 weeks	Phase I single-arm clinical trial	Standard chemoradiation	Primary focus was safety and feasibility, with PFS, OS, QoL and cognitive outcomes assessed as secondary endpoints	Single-arm design and small sample; not powered to establish efficacy	Early clinical
**Ovarian / endometrial cancer**	High-fat, low-carbohydrate KD	Randomized controlled trial	Chemotherapy	Demonstrated feasibility of dietary intervention and metabolic/body-composition effects	Small sample and relatively short intervention; oncologic efficacy was not the primary endpoint	Early clinical
**Breast cancer**	KD / carbohydrate-restricted ketogenic protocol	Randomized and pilot clinical studies	Chemotherapy or standard treatment	Changes in metabolic parameters and body composition have been reported; some studies suggest possible effects on treatment-related outcomes	Heterogeneous protocols, limited sample sizes and variable adherence	Early clinical
**Pancreatic cancer**	Ketogenic/metabolically restricted dietary approaches	Early clinical studies / ongoing trials	Chemotherapy	Feasibility and metabolic effects have been investigated; definitive evidence for improved survival remains unavailable	Small cohorts and incomplete outcome data	Investigational
**Head and neck cancer**	KD during radiotherapy/chemoradiotherapy	Pilot randomized/controlled study	Radiotherapy or chemoradiotherapy	Primarily evaluated body composition, nutritional and metabolic outcomes rather than definitive tumor-control endpoints	Small sample and short duration	Early clinical
**Mixed solid tumors**	Various KD protocols	Prospective and observational studies	Different anticancer therapies	Clinical studies demonstrate that KD can alter glucose/ketone metabolism and is feasible in selected patients; antitumor effects remain inconsistent	Heterogeneous cancer types, treatments and dietary protocols	Low / heterogeneous
**Immunotherapy combinations**	KD combined with immune checkpoint therapy	Emerging translational/clinical studies	Anti-PD-1/PD-L1 or other ICI	Strong mechanistic rationale and emerging clinical interest, but definitive evidence for improved response, PFS or OS remains limited	Few adequately powered randomized trials	Preliminary / investigational

## Future directions

### Rethinking carbohydrate effects / diet basis

Carbohydrate reduction in cancer needs to be discussed as part of the larger framework of tumor metabolism rather than being viewed as an inherently good intervention. KD causes a reduction in carbohydrates and induces nutritional ketosis, leading to alterations in circulating glucose, insulin, fatty acids, and ketones [12, 20]. Such effects can cause increased metabolic stress in tumor cells which are heavily reliant on glycolysis and cannot adapt metabolically, whereas adaptable tumors may find alternatives in oxidative phosphorylation of fatty acids or utilization of glutamine, among others [12, 20].

It is important to note that the effects of carbohydrate reduction are not automatically selective against tumor cells. Even though reduced glucose concentration might negatively affect highly glycolytic tumors, glucose is essential for activation of immune cells. Hence, carbohydrate reduction may potentially harm immune function while ketone utilization might help adaptable immune cells. Such interplay of effects will depend on types of both tumors and immune cells, nutritional and disease conditions. Research on the effects of fasting, fasting-mimicking diets, glucose restriction, or exogenous ketone administration needs to be differentiated from KD-based research [46].

Therefore, the currently available data does not provide a justification for viewing KD as inherently anti-carcinogenic or pro-tumor. The available human evidence is heterogeneous and has not revealed any improvement in tumor control and overall survival [12, 20]. “Rethinking carbohydrate effects” implies considering carbohydrate availability as a part of the larger metabolic environment, instead of assuming that carbohydrate reduction per se is responsible for cancer development. Hence, KD needs to be studied as an accessory to metabolic therapy.

### Biomarker-guided ketogenic diets

Scientists have been working on designing specific ketogenic diets based on their respective biomarkers [10, 29]. Every tumor has developed its own mechanism of procuring energy through different sources, such as sugar. Based on this research, ketogenic diets may be effective for some but not all. Researchers need to identify biomarkers, such as signs related to tumor metabolism, immune system activity, mitochondrial function, or gut bacteria, to predict which patients are most likely to benefit from the diet [6, 10]. These synthetic biomarkers may help in diagnosis and also tracing the progress of treatment.

### Precision metabolic oncology and combination therapy

Ongoing research on metabolic oncology can plausibly enhance the role of the ketogenic diet (KD) on the therapeutic effects of cancer treatment. Rather than using the same ketogenic approach for all patients, future treatments may be customized according to the unique biological features of each tumor. By studying a patient’s genes, metabolism, and immune system, doctors may be able to identify which tumors are more sensitive to low glucose levels or ketone bodies [10, 47]. This could turn the ketogenic diet from a general dietary approach into a personalized metabolic strategy within precision cancer care.

A major opportunity is the combination of KD with immunotherapy and targeted treatments. Evidence suggests that a ketogenic environment may improve T-cell function, reduce immune exhaustion, and enhance anti-tumor immune responses. Because of this, KD may potentially improve the effectiveness of therapies such as immune checkpoint inhibitors and CAR-T therapy [8, 12]. Similarly, combining KD with drugs like mTOR inhibitors, metformin, or IDH inhibitors may increase metabolic stress on cancer cells by blocking multiple survival pathways at once [31, 40]. Therefore, future research should not focus only on KD alone, but also on its role in combination therapies to improve cancer outcomes.

### Personalized implementation and future research needs

In the future, greater emphasis should be placed on developing personalized ketogenic diets tailored to individual patient needs, treatment regimens, and clinical conditions. A single standardized ketogenic plan may not be suitable for all patients, especially those with cachexia, malnutrition, or advanced disease [35, 40]. Considering calorie requirements, tumor biology, and treatment intensity, individualized nutritional planning may improve both safety and effectiveness. Emerging tools such as digital health monitoring, continuous metabolic tracking, and microbiome analysis may further support personalized ketogenic interventions.

Importantly, future research must also address current limitations through well-designed clinical studies. It is essential to better understand how KD affects cancer through metabolism, immunity, gut microbiota, and gene regulation. Overall, KD is likely to remain a supportive strategy that enhances, rather than replaces, existing cancer therapies [10].

## Conclusion

The ketogenic diet (KD) is increasingly being recognized as more than a simple metabolic intervention in cancer therapy. Emerging evidence suggests that KD may function as a systems-level immunometabolic sensitization strategy capable of enhancing therapeutic responsiveness through interconnected biologic mechanisms. By simultaneously inducing metabolic stress, modulating immune activity, remodeling the microbiome and influencing epigenetic regulation, KD may create a coordinated biologic environment that supports improved treatment sensitivity [8, 20, 22].

Importantly, the potential value of KD may not lie in directly eliminating malignancy as a standalone therapy, but rather in amplifying the effectiveness of existing treatment modalities such as chemotherapy, radiotherapy, immunotherapy and targeted metabolic agents [5, 28]. This systems-level perspective shifts KD from an isolated nutritional intervention toward a broader therapy-enhancement platform in which metabolic and immune pathways mechanistically converge.

Nevertheless, current evidence remains preliminary and heterogeneous. Significant variability exists across tumor types, patient populations and clinical study designs, and many mechanistic interactions remain incompletely understood [9, 14]. Therefore, further mechanistic investigations and well-designed prospective clinical trials are necessary to determine optimal patient selection, therapeutic combinations and long-term safety.

Overall, KD represents a promising emerging strategy within precision metabolic oncology. Continued research integrating metabolism, immunity, microbiome biology and therapeutic response may help define its future role as a coordinated adjunctive approach for improving cancer treatment outcomes.

## Data Availability

No datasets were generated or analysed during the current study.
